# Clinical Society of Maryland

**Published:** 1894-06

**Authors:** H. O. Reik

**Affiliations:** Secretary; 525 N. Howard Street; Baltimore


### Society Reports.

---

### CLINICAL SOCIETY OF MARYLAND.

BALTIMORE, March, 1894.

The 292d regular meeting was called to order by the President, DR. J. E. MICHAEL.

DR. G. J. PRESTON read a paper advocating the establishment of detention wards for cases of suspected insanity.

DR. BILLINGSLEA spoke of a case which had recently come under his notice where a lady of respectable family became suddenly deranged and was arrested while in a city store and confined for two days, first in the station-house and then in the city jail, before being sent to an asylum. He thought that detention wards were needed for such cases.

DR. JOHN MORRIS thought that there was a great necessity in Baltimore for a detention hospital or ward. He did not think that the station house or jail or general hospital was a fit place for cases of suspected insanity.

DR. G. H. ROHE agreed with Dr Preston as to the necessity of detention wards, and spoke of a class of cases not mentioned by Dr. Preston, namely, persons pronounced insane by examining physicians but who at present must be kept at home until arrangements can be made to transfer them to some institution, often with great inconvenience and even danger to the relatives.

DR. PRESTON hoped that some hospital would inaugurate such wards, and believed that the city and State would aid in the construction and maintenance of the ward.

DR. R. S. NORMENT read a paper on "The Management of Typhoid Fever in Private Practice among the Poor and Middle Classes."

Under the head of "General Management" he stated that there was no abortive treatment. As a general rule it is best to let the patient know his condition, as this induces the patient to take proper care of himself in the early stages. As soon as the disease is recognized, the patient should be put to bed in a comfortable, quiet, well ventilated room. The patient and nurse should be the only occupants of the room. There should be but little body clothing and the bed linen and body clothing should be changed frequently. If the abdominal symptoms are not violent, the patient can be helped into a chair for a short time daily. The body should be kept clean by sponging with tepid water every twenty-four to forty-eight hours.

*Diet:* Milk, beef tea, mutton broth, raw eggs, barley and rice water, are better than the artificial foods of the stores. It is not best to insist on the taking of food in the early stages. Water should be given freely, but not in sufficient quantity to over-distend the stomach.

*Medicine:* The average American must be medicated when he is sick. As a routine practice the author gives one and a half to two grains of quinine with dilute muriatic acid every four hours.

*Alcohol:* When weakness is very pronounced and there is hypostatic condition of the lungs, the free administration of whisky is of great service.

*Temperature:* If the temperature does not exceed 103° and is the only pronounced symptom, it is better to let it alone; above this, the bath in one form or another is best. The author's experience is limited to the sponge-bath. An oil cloth is put on the bed and water at 90° F. is applied with a good sponge. The patient is sponged until it ceases to be agreeable to his feelings. Tincture of aconite and bromide of potash, or antipyrine in five grain doses can be given when the temperature exceeds 103°. Phenacetine is very depressing and should be used with caution—five grain doses three or four hours apart. Tincture of aconite root, one to three drops, and bromide of potash, ten grains, may be given every two or three hours, when there is a hard pulse and delirium. Antipyrine is the best antipyretic where headache is the principal accompaniment of high temperature. When the patient is maniacal, chloral is indicated. For stupor, maintain the proper action of the bowels and kidneys and give alcohol to keep up the heart's action.

*Diarrhea:* If stools fewer than five or six, no treatment necessary; if more, then give bismuth and an astringent or tincture of opium by the bowel.

*Hemorrhage:* Absolute rest; gallic acid and opium freely; cracked ice for thirst.

*Perforation:* He has never seen a patient with perforation recover.

*For Pain:* Morphia.

*Recovery:* No solid food should be given until the fever is below 99° for two days. The patient should not be allowed to sit up until the temperature is below 99° and only for an hour or so at first.

DR. OSLER said he would like to be under Dr. Norment's treatment if he had an uncomplicated case of typhoid fever without pyrexia. He thought the antipyretic drugs were entirely superfluous in this disease. The cold bath is more efficacious, but is not always available in private practice; but all the good effects of the bath can be obtained by sponging. A good nurse or doctor can sponge the patient so effectually that the fever will be satisfactorily reduced. When the temperature is high, ice-sponging—not with ice water but with lumps of ice—over the back and legs will reduce the temperature very pleasantly to the patient and satisfactorily to the doctor. Delirium and stupor are also effectually treated by ice sponging. The use of the modern antipyretics in typhoid fever is in nine cases out of ten positively hurtful; they reduce the heart's action and cause weakening sweats and their use is an unmitigated evil. In the great majority of cases, the treatment may be taken from old Dr. Nathan Smith, of Yale, which was pretty much that of to-day: Plenty of fresh air, liquid diet, and cold externally. He was in the habit of turning out the friends of the patient, putting the patient on the floor and then dashing water, handed through the window by an assistant, over the patient.

DR. WINSLOW did not believe that it was necessary to give medicine in typhoid fever, except in special cases. Before using the cold bath system he had used antifebrine and was pleased with the results. It gave the patient comfort and no bad results were observed.

DR. G. J. PRESTON, speaking of the coma of typhoid fever, said that while cold baths were best for this condition, yet in some instances it did not relieve it. Strychnine and caffeine used hypodermically have given good results. Recently he had seen very good and prompt antipyretic effects from the use of 20 to 30 drops of guaiacol applied externally.

DR. PEARCE KINTZING said that he could not overcome the prejudice of people against cold water. He uses antifebrine with quinine. With this he secures a reduction of temperature for five or six hours. After cold sponging the temperature comes up quicker.

DR. J. M. CRAIGHILL had never had any trouble in getting his patients sponged with cold water when a little vinegar was added to the water. In the better class of patients he had alcohol added to the water.

DR. C. H. MITCHELL had had considerable experience with typhoid; he has used antipyretic drugs and his results are as good as those reported by those who have not used them. The majority of cases which he loses are not those having high temperature. Phenacetine is by far the best antipyretic in his experience. He uses it in small doses, two and one-half grains every four hours. He gives tonic doses of quinine in the early part of the day and two and one-half grain doses of phenacetine until midnight. It is impracticable in private practice among the poorer classes to use the bath. One of his patients, a child, was made worse by the fright which occurred every time the bath was used. Deaths are not due to high temperature and occur in those cases where a bath would not have saved them.

DR. NORMENT believed that in this part of the country amongst the poorer classes, the prejudice against the use of the bath in disease was insurmountable. Under these circumstances, some of the antipyretic drugs occasionally do good.

DR. OSLER, replying to a question of Dr. Norment, said that he did not believe in the use of alcohol as a routine practice, but in such cases as Dr. Norment had referred to, where there was pronounced weakness and in hypostatic congestion, the use of whisky was very valuable. He preferred it in combination with strychnia.

DR. JOHN MORRIS then introduced the following resolution:

*Resolved,* That in furtherance of public order, public decency and humane sentiment, execution by electricity should be substituted for hanging as the death penalty for crime.

DR. J. E. MITCHELL was opposed to the resolution. He did not believe that the death by electricity was any more rapid or less painful than by hanging. He was heartily in favor of capital punishment, and thought hanging preferable to any other mode of execution, for it was not complicated and not bloody.

DR. NORMENT was not opposed to capital punishment; but he was opposed to electrocution. He thought that death by chloroform was advisable, being painless and cheap.

DR. G. J. PRESTON was opposed to capital punishment, but as long as capital punishment existed he was in favor of hanging and opposed to electrocution. He had seen one person hung, and he thought that electrocution could not equal it for humanity.

After further discussion the resolution was lost by an almost unanimous vote.

---

BALTIMORE, MD., April, 1894.

The 294th regular meeting of the Clinical Society of Maryland was called to order by the Vice-President, DR. HERBERT HARLAN.

DR. G. L. TANEYHILL read a paper entitled "Notes on and Personal Experience with Puerperal Mania."

DR. JULIAN FRIEDENWALD then read a paper on the "External Use of Guaiacol as an Antipyretic," comprising work done in conjunction with Dr. H. H. Hayden.

"This drug was applied in seventeen cases, eight cases of pneumonia, two cases typhoid fever, two cases pulmonary tuberculosis, one case acute articular rheumatism, one case facial erysipelas.

Their conclusions are:

1. That this drug has a powerful antipyretic action occasioning a reduction of from one to four degrees of temperature in from one to four hours.

2. That in all cases this reduction of temperature is accompanied by profuse diaphoresis, which may or may not be accompanied by a chill or chilly sensation.

3. That great exhaustion is frequently produced.

4. That the effects may be obtained from comparatively small doses (from thirty to fifty drops), and that great care should therefore be exercised in the use of the drug. The drug should be applied but once or twice daily and the initial dose should not be above thirty drops.

5. That the effect produced by guaiacol, though more powerful, is the same as is obtained from most of the other antipyretics of the coal tar series and that the same care must therefore be exercised as with the other preparations. Its effect differs from the stimulating cold bath in being *depressant*.

6. That the main indication for its use is in diseases accompanied by high fever, in which the cold bath cannot be applied. It may therefore be especially useful in typhoid fever, as well as in all other diseases accompanied by high fever, in which irritability of the stomach prevents the use of other antipyretics.

DR. KINTZING: It seems Dr. Friedenwald did not vary the dose. Perhaps he might have received the same fall in temperature in some of his cases on smaller doses and had less depression.

DR. FLEMING: I have tried the drug in two cases and had such depression in both that I decided not to use it further.

DR. NORMENT: I see two troubles in regard to the use of guaiacol. First the depression is very sudden, and second, the reaction is violent. It seems to me from Dr. Friedenwald's paper that guaiacol is of very questionable value in private practice.

---

BALTIMORE, April, 1894.

The 295th regular meeting of the Clinical Society was called to order by the President, Dr. J. EDWIN MICHAEL.

DR. W. E. MOSELY read a paper on the "Importance of a Microscopical Examination in the Differential Diagnosis of Diseases of the Uterus," treating the subject from a clinical standpoint.

DR. W. T. HOWARD, JR., followed with a paper on the same subject from a pathological point of view.

DR. H. HARLAN read a paper on "Cases of Foreign Bodies in the Eye, associated with Sympathetic Ophthalmia."

The first was that of a young man, aged thirty-seven, who had received a blow in the eye from a piece of the head of a nail fifteen years previously. He consulted me because the good eye had recently been growing misty. Ophthalmoscopic examination showed irido-plegia and slight neuritis with the dimness of vision, and, regarding the trouble of sympathetic origin, I advised enucleation of the lost eye. The bit of steel was found just posterior to the ciliary bodies.

Case second was a farmer aged thirty-eight who came with the eye red and inflamed, without light perception and painful to the touch. History was, that seven years previous, while passing through a woods, he had been struck in the eye by a branch of a sweetgum tree. Eye had been more or less painful ever since, at times very painful. The sound eye had never given any trouble. After enucleation the eyeball was opened, and just behind the iris was found an entire small terminal bud, conical in shape, and covered by the hard shell that is found over the buds of sweetgum in late winter.

Here we have two cases where a foreign substance remained in an eye many years, one fifteen and the other seven. One a particle of steel, which possibly entered in an aseptic condition; the other a fresh growing vegetable fiber. One remaining quiescent for fifteen years, and then setting up sympathetic trouble in the good eye; the other probably carrying many organisms and causing nearly constant irritation for seven years, and causing no trouble whatever in the sound eye.

Dr. Harlan then considered at some length the history of sympathetic ophthalmia, its causes and the various theories as to how it is brought about. He was rather inclined to take issue with Deutschman, and to support the Ciliary Nerve Theory, concluding as follows: "We must admit that, notwithstanding a great deal of well-directed experimental work, little has been accomplished towards the solution of the problem of sympathetic ophthalmia. The fact that its infectious origin has not yet been clearly proven is to my mind one of the strongest arguments against its being of that nature. If infectious, micro-organisms ought to be present always, and frequently found in eyes taken out for causing sympathetic trouble in the fellow eye. Again, if infectious, by whatever means the infection spreads, whether by way of nerves, blood vessels or lymphatic channels, there is no satisfactory explanation of the disease being so strictly a local one."

DR. HIRAM WOODS said that the nature of sympathetic ophthalmia seemed as mysterious as ever. He did not think that Deutschman's explanation had been completely overthrown. His researches, as well as those of Alt and Gifford, had proved that infection of one can involve the other eye; that the path lies most probably along the lymph channels of the optic nerve, the orbital vessels, etc. Negative cannot always set aside positive testimony. In this instance it has proved that infection of one does not necessarily involve the other eye—possibly does so only rarely. The well-known frequency of sympathetic ophthalmia after panophthalmitis is a bit of clinical evidence against the liability of an eye to become inflamed when its fellow is suffering from an infection. Dr. R. L. Randolph was quoted as authority for the statement that organisms are rarely found in an eye enucleated after sympathetic disease has started in the other. All this proves that there must be some other explanation than infection, but does not disprove the possibility of the latter against well authenticated positive results.

Dr. Woods narrated two cases of sympathetic ophthalmia following injuries which did not destroy sight. In one patient both eyes were ultimately destroyed by the plastic irido-cyclitis, light perception alone remaining. In the other the injured eye eventually retained better vision than the other.

H. O. REIK, M.D., Secretary.

525 *N. Howard Street.*

